# Management of Recurrent Metastasis of Renal Cell Carcinoma to the Parotid Gland After Nephrectomy

**DOI:** 10.1002/ccr3.70765

**Published:** 2025-10-05

**Authors:** Kathryn Collins, Emily Cushing, Alex Ellerhorst, Christopher Selinsky

**Affiliations:** ^1^ Department of Otolaryngology/Head and Neck Surgery OhioHealth Doctors Hospital Columbus Ohio USA; ^2^ Ohio University Heritage College of Osteopathic Medicine Dublin Ohio USA; ^3^ Department of Otolaryngologayl OhioHealth Doctors Hospital Columbus Ohio USA

**Keywords:** non‐primary parotid malignancy, recurrent metastases after nephrectomy, renal cell carcinoma, same sided recurrence

## Abstract

Renal cell carcinoma accounts for 90% of malignant renal tumors, with common metastases to the lungs, bones, and brain. Head and neck metastases are uncommon, and the parotid gland is an exceedingly rare site (0.5%). Recurrent metastasis to bilateral parotid glands has been reported in the literature, but recurrent metastasis to the ipsilateral gland has not. This is a case about a 69‐year‐old female who presented to our clinic with the chief complaint of a slowly enlarging left neck mass. She had a history of clear cell renal cell carcinoma treated with nephrectomy 15 years prior. An MRI reviewed from 2 years earlier incidentally noted a left parotid tail lesion that had not been evaluated. CT and fine needle aspiration biopsy raised concern for malignancy. The patient underwent superficial parotidectomy, which confirmed metastatic clear cell renal cell carcinoma. Surveillance imaging was recommended but not completed. Two years later, she re‐presented with recurrence in the same parotid gland. A multidisciplinary tumor board recommended surgery with preservation of the facial nerve, followed by radiation and immunotherapy. This case underscores the unpredictable metastatic pattern of renal cell carcinoma and the importance of long‐term surveillance even beyond recommended guidelines. Clinicians should maintain a high index of suspicion for metastatic renal cell carcinoma in patients with a history of nephrectomy presenting with a parotid mass, given the potential for late recurrence.

**Summary**

- Recurrent metastatic clear cell renal cell carcinoma (ccRCC) to the same parotid gland is exceedingly rare. To our knowledge, no cases have been previously reported in the literature.
- This case highlights the necessity of long‐term surveillance post‐nephrectomy and the importance of considering metastatic RCC in patients presenting with parotid masses, even years after initial treatment.

## 1 Introduction

Renal cell carcinoma (RCC) accounts for 90% of malignant renal tumors [1]. RCC is known for its unpredictable nature and hematogenous spread, commonly to the lungs, bones, and brain [1]. Head and neck metastases are rare, but the thyroid gland is the most common location, accounting for 5%–6% of all RCC metastases. The parotid gland is among the least common. Recurrent metastasis to the ipsilateral parotid gland is exceedingly rare, and based on current literature review, there are no reported cases of this occurrence; previously documented cases have only described recurrent metastasis to the contralateral parotid gland [2, 3]. A recent systematic review by Gupta et al. confirmed that parotid metastasis from RCC remains exceedingly rare, accounting for less than 1% of cases, and emphasized the potential for delayed presentations many years after initial nephrectomy [2].

This case report highlights the clinical presentation, diagnostic findings, and management of a patient with recurrent metastatic clear cell renal cell carcinoma (ccRCC) to the parotid gland, 15 years after nephrectomy.

## 2 Case History/Examination

A 69‐year‐old female presented to the otolaryngology clinic with an asymptomatic, enlarging, left‐sided neck mass present for 3 months. Her history was significant for ccRCC treated definitively with nephrectomy 15 years prior, with no follow‐up visits or imaging since surgery. Additionally, her past medical history included anxiety, depression, migraine, arthritis, carpal tunnel syndrome, and hypertension. Past surgical history consisted of nephrectomy, cholecystectomy, dilation and curettage, knee arthroplasty complicated by meniscal tear, and open reduction of the wrist. Laboratory results were equivocal. The physical examination was notable for a non‐tender, 3‐cm firm mass in the left level II neck space. Upon chart review, a 1.7‐cm left‐sided mass in the parotid tail was evident on magnetic resonance imaging (MRI) of her cervical spine obtained 2 years prior for a fall. There was no formal evaluation of this mass following the imaging.

## 3 Methods

**FIGURE 1:**
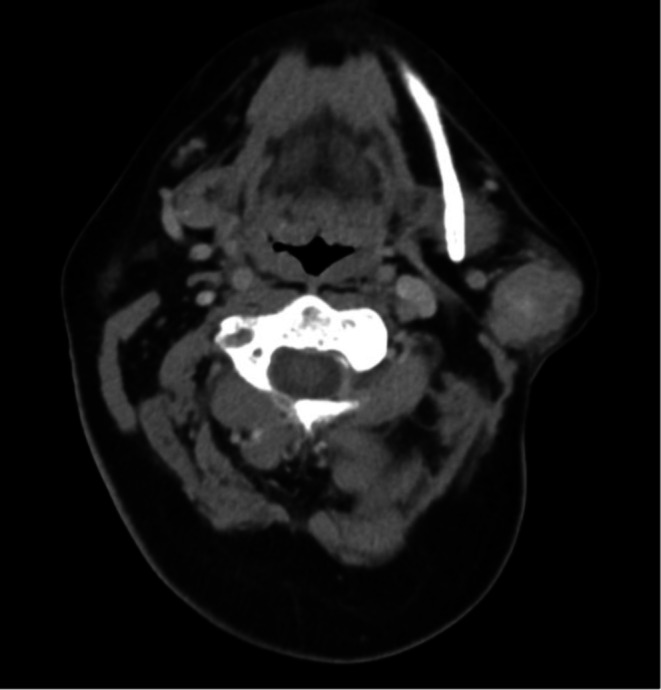
CT soft tissue head and neck displaying parotid tail lesion.

A computed tomography (CT) scan of the neck and fine needle aspiration (FNA) biopsy of the mass were obtained. Imaging revealed a left parotid tail mass measuring 30 x 25 x 26 mm with lobulated areas of focal enhancement (Figure 1). FNA demonstrated cohesive cell clusters with fibrovascular cores, finely granular to bubbly cytoplasm, and round nuclei. The specimen was graded as high‐grade cytologic atypia with unknown significance.

**FIGURE 2:**
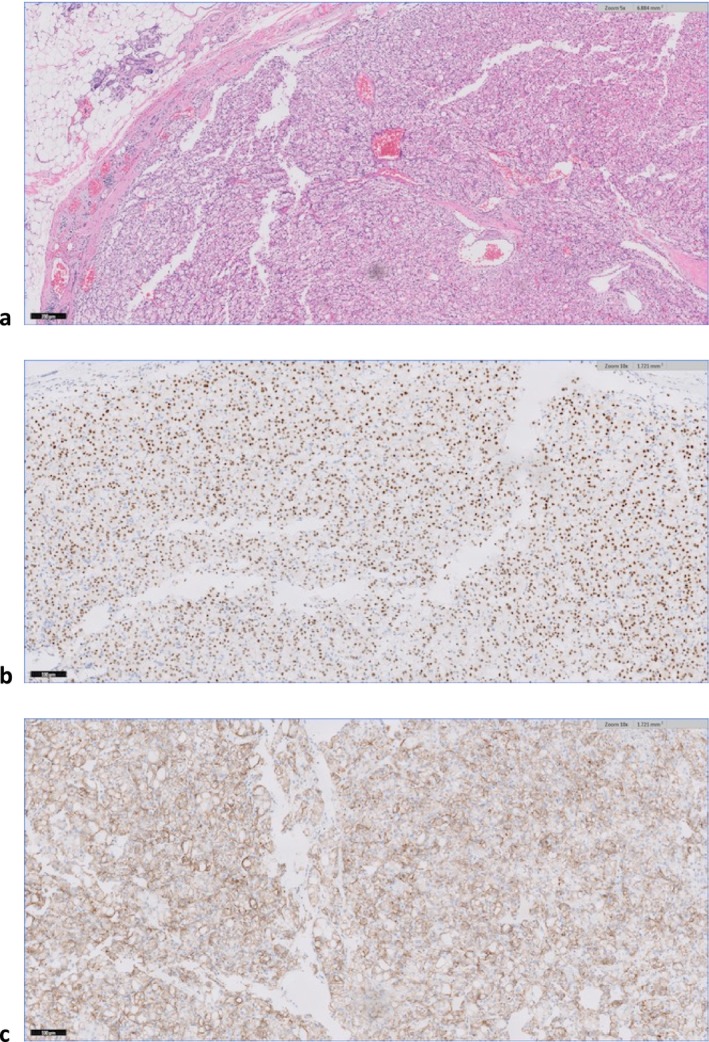
(a) H&E staining of surgical pathology of parotid lesion. (b) PAX‐8 positive immunohistochemical staining of parotid lesion. (c) CAIX positive immunohistochemical staining of tail of parotid lesion.

The patient underwent superficial parotidectomy, and the intact mass was excised. Pathology revealed tumor cells with abundant clear cytoplasm, distinct cell borders, and an intricate vascular network that was positive for PAX‐8, carbonic anhydrase IX, and CD10 (Figure 2). Findings were consistent with metastatic ccRCC.

Following discussion during a multidisciplinary tumor board, a CT of the chest, abdomen, and pelvis, along with a technetium‐99 bone scan, was obtained. No concerning lesions were found. A consensus decision was that no further adjuvant therapy was necessary, and surveillance with a CT neck in 1 year was recommended. The patient was noncompliant and lost to follow‐up until she re‐presented 2 years later with a new, asymptomatic left‐sided neck mass, present for 7 months. This mass was nearly identical in location and appearance to the previously excised tumor.

A CT scan of the head and neck, and FNA of the mass were obtained. Imaging showed a left superficial parotid lobe mass measuring 32 x 28 x 25 mm. FNA confirmed a clear cell neoplasm consistent with previously diagnosed metastatic ccRCC. A positron emission tomography (PET) scan was obtained and confirmed a mildly hypermetabolic left parotid mass consistent with metastatic disease, with no additional areas of hypermetabolic activity.

Her case was again discussed at the multidisciplinary tumor board. The goal was to treat the recurrence aggressively while limiting injury to her facial nerve. The recommended intervention included revision parotidectomy with preservation of the facial nerve, followed by adjuvant radiation and immunotherapy. During the surgery, the mass was adherent to portions of the facial nerve and intricately involved with the retromandibular vein. The majority of the mass was removed; however, a small portion of the tumor capsule was left behind to preserve the integrity of the nerve.

## 4 Conclusions and Results

Following surgery, complete loss of facial nerve function was present. She subsequently underwent radiation therapy to the wound bed and deep parotid lobe using an intensity‐modulated radiation therapy (IMRT) technique, receiving a total dose of 30 Gy in 10 fractions over 2 weeks. Over the next 4 months, her facial nerve function gradually improved to near preoperative baseline (House‐Brackman II/VI). Further treatment will include pembrolizumab (Keytruda) with the hope of complete long‐term remission.

## 5 Discussion

Clear cell renal cell carcinoma (ccRCC) is characterized by unpredictable metastatic patterns, often manifesting years after initial nephrectomy, making diagnosis challenging [1, 2]. According to the American Urological Association (AUA) guidelines, routine surveillance imaging is recommended for at least 5 years following nephrectomy, with continued long‐term monitoring based on individual risk factors [4]. In this case, the patient had no follow‐up imaging for over a decade, underscoring the importance of long‐term surveillance to detect potential metastases before symptomatic progression.

Notably, the recurrence of metastatic ccRCC to the same parotid gland is unprecedented in the literature. In their systematic review, Gupta et al. found no reported cases of ipsilateral parotid gland recurrence, with most documented cases involving solitary or contralateral lesions [2, 5]. The National Comprehensive Cancer Network (NCCN) guidelines for post‐nephrectomy surveillance for RCC vary by stage [6]. For Stage I, CT/MRI chest and abdomen should be completed 3–12 months post‐surgery, followed by annual imaging for 5 years, with increasing frequency for higher‐stage disease [4, 6]. While recurrence is most common within 5 years, head and neck metastases often follow a different timeline. This case presentation highlights the importance of recognizing ccRCC's highly variable metastatic behavior and incorporating long‐term surveillance strategies.

The management of metastatic RCC to the parotid gland poses significant diagnostic challenges. FNA is often used for initial evaluation of a parotid mass; however, distinguishing metastatic RCC from primary salivary gland tumors can be challenging [3, 7]. Core needle biopsy (CNB) provides greater architectural detail and improves diagnostic accuracy [7]. In this case, FNA raised suspicion for malignancy but was inconclusive, necessitating surgical excision for histopathologic confirmation. A prior study comparing FNA to CNB found that CNB had a higher diagnostic success rate, with all six CNB samples confirming metastatic RCC, compared with only one out of nine FNA samples [7]. Similarly, in our case, both initial FNAs were non‐diagnostic, prompting surgical intervention.

Traditionally, RCC has been considered radioresistant, but recent studies suggest that stereotactic body radiation therapy (SBRT), which delivers high‐dose radiation in a few fractions, can provide effective local control of gross lesions [8]. In this patient, postoperative IMRT was used to treat microscopic disease in the wound bed following re‐excision. This conventional fractional approach allowed for precise targeting while minimizing exposure to the facial nerve and surrounding healthy tissue.

Metastatic renal cell carcinoma should be included in the differential diagnosis in patients with a history of RCC presenting with parotid masses for the following reasons: an extended latency period, the ability to mimic primary malignancy and benign neoplasms on imaging, propensity for misdiagnosis, and generally asymptomatic presentation. Surgical excision with facial nerve preservation followed by IMRT may lower the risk of facial nerve injury by eliminating the need for re‐excision while maintaining nerve function, emphasizing the need for further research to assess safety and long‐term efficacy.

## Author Contributions

**Kathryn Collins:** conceptualization, resources, supervision, writing – original draft, writing – review and editing. **Emily Cushing:** conceptualization, data curation, writing – original draft, writing – review and editing. **Alex Ellerhorst:** writing – original draft. **Christopher Selinsky:** conceptualization, project administration, resources, supervision, validation, writing – review and editing.

## Consent

Written and verbal informed consent was obtained from the patient.

## Conflicts of Interest

The authors declare no conflicts of interest.

## Data Availability

The data that support the findings of this study are openly available at http://doi.org/10.1017/s0022215100112691.
